# Demographic factors, partial social belonging and psychological resources associated with coping

**DOI:** 10.3389/fpsyg.2023.1154659

**Published:** 2023-04-03

**Authors:** Yohanan Eshel, Shaul Kimhi, Hadas Marciano, Bruria Adini

**Affiliations:** ^1^Stress and Resilience Research Center, Tel-Hai College, Tel Hai, Israel; ^2^Department of Psychology, University of Haifa, Haifa, Israel; ^3^ResWell Research Collaboration, Tel Aviv University, Tel Aviv, Israel; ^4^The Institute of Information Processing and Decision Making, University of Haifa, Haifa, Israel; ^5^Department of Emergency and Disaster Management, School of Public Health, Sackler Faculty of Medicine, Tel Aviv, Israel

**Keywords:** perceived partial social belonging, societal resilience, individual resilience, coping indicators, psychological symptoms

## Abstract

**Introduction:**

The present study investigates the role of perceived partial social belonging (PPSB) in determining societal and individual resilience and positive and negative coping indicators. It is assumed that most people aspire to belong and be integrated into their society. A sense of only partial belonging is therefore distressing for them.

**Methods:**

Two hypotheses are examined in the current study: (a) A higher level of PPSB will predict a lower level of resilience and a higher level of psychological symptoms. (b) PPSB will mediate the associations between three stress-evoking demographic characteristics (younger age, low income, and gender) and the lower psychological resilience and higher distress associated with these demographic characteristics. These hypotheses were examined using a sample of the Israeli Jewish public (*N* = 1,502) who responded to an anonymous questionnaire about the investigated issues. The data were collected by an internet panel company possessing a database of more than 65,000 residents, representing the varied components of the Israeli society.

**Results:**

The findings supported our hypotheses: (a) PPSB negatively predicted societal and individual resilience and hope and positively predicted distress symptoms and sense of danger. (b) PPSB mediated the effects of the investigated demographic variables on these psychological variables.

**Conclusion:**

These results are discussed in association with the concept of belonging competencies. Our findings display that being unsure about one’s belonging to a desired social group, has a major role in increasing psychological distress and sense of danger and in reducing hope and both individual and societal resilience.

## Introduction

The present study examines a rather new issue that was not investigated thoroughly in the social psychology research on belonging: the state of perceived partial social belonging (PPSB), its demographic determinants, and psychological impacts. Sociological perspectives have defined social integration and segregation in behavioral terms, that is by individual and group participation in social relationships with other group members, and by their success in achieving social cohesion (Hirsch et al., 2008; Knight and Eisenkraft, 2015). Partial social integration has been similarly discussed in cases of partial exclusion of individuals or groups from full participation in the society in which they live (Rawal, 2008). In contrast to the concepts of integration and segregation perceived partial social belonging (PPSB) does not refer to an objective criterion of belonging but rather to the sense of individuals that they do not fully belong to their society, community or group and to their doubts whether and to what extent are they accepted as full members by other members of this society (Eshel et al., 2022b). PPSB can be felt in any social setting despite a policy of integration and public declarations of social acceptance, and is likely to be associated with decreased wellbeing and resilience as well as with enhanced distress.

Deci and Ryan (2000) claim that most people seek to satisfy the need for continued positive relationships with others since “the desire for interpersonal attachments–the need to belong–is a fundamental human motivation,” (Baumeister and Leary, 1995, p. 520). The sense of belonging supports wellbeing and other positive psychological outcomes (Cacioppo et al., 2015; Erzen and Çikrikci, 2018). Deficient satisfaction with this need to belong is likely to cause frustration and interrupt the health and resilience of individuals and communities worldwide (Allen et al., 2021).

Earlier studies analyzed the efforts of groups to belong in terms of social integration. Sociological perspectives often describe interactions between two groups in terms of integration compared to segregation (Ziller and Spörlein, 2020). Integration is aimed at eventually creating a mutual group out of the two different groups, whereas segregation is marked by efforts to separate these two groups and to prevent mutual social interaction between their members. Research shows that segregation raises negative emotions and low levels of wellbeing and resilience among the members of the group that wish to belong and be accepted (Ferdinand et al., 2015). Berry’s (1997) analysis of belonging claims that immigrants are faced with four alternative acculturation strategies, reflecting their level of identification with their original ethnic culture compared to the majority’s culture. To achieve the state of belonging or integration, they should find a way to develop a strong identification with both the original and the majority culture. According to this theoretical position, belonging is determined mainly by the newcomers’ choices with little input from the general society. More recent analyses claim that the two-way process of belonging (e.g., Klarenbeek, 2021) exists “because of and in connection with the systems in which we reside” (Kern et al., 2020, p. 709).

Social belonging that is expressed by social identification and social cohesiveness reflects the extent to which the participating individuals regard themselves as absorbed in their society (Knight and Eisenkraft, 2015). However, there is a disagreement concerning the road to belonging. One theory suggests that it is determined by the number of social interactions, active participations, and social roles, such as a friend or a volunteer, carried out by the individual (Turner and Turner, 1999; Baumgartner and Susser, 2013; Chin et al., 2018). A second position refers to belonging in terms of the psychological experience of those who wish to belong (Klarenbeek, 2021). This experience is a basis for a sense of control, competence, and wellbeing (Antonovsky, 1979; Cowen, 2000; Hirsch et al., 2008). It has been found that denying the aspiration to belong has a lasting negative impact on social interaction and cooperation (Gracia and Herrero, 2004; da Costa et al., 2020).

A recent analysis of belonging emphasizes an individualistic perspective on belonging, defined as a subjective feeling of being an integral part of one’s family, friends, work environments, communities, cultural groups, and physical places (Allen, 2020). Allen et al.’s (2021) theoretical model conceptualizes the construct of belonging as a dynamic experience composed of four interrelated components: competencies, opportunities, motivations, and perception. Most people share social competencies of belonging, which are used for interacting with others, developing a sense of identity, and connecting their cultural experience with their country. These competencies are central to their social and emotional learning (Durlak et al., 2011). Belonging competencies are useless in the absence of opportunities to connect or in the face of barriers to belonging. These opportunities, that include accessible groups, people, places, times, and spaces, are determined by the desired groups’ readiness to enable inclusiveness or to enforce exclusiveness (Allen et al., 2019; Roffey et al., 2019). Motivation to belong refers to seeking positive interactions with others and transforming them into long-term relationships (Baumeister and Leary, 1995). These authors emphasize that the motivation to belong differs from attachment since it is not aimed at a specific individual but at significant social relationships in general. The fourth component of this theoretical model is perceived belonging or people’s evaluation of how much they belong or blend socially (Walton and Brady, 2017). Allen et al. (2021) model claims that connecting skills, opportunities, and a motivation to belong depend on the individuals’ perception of belonging and the meanings they assign to their capacities, options, and incentives to belong and to interact with others (Lambert et al., 2013).

### Belonging in the Israeli context

The vast majority of the Jewish population in Israel constitutes the second or third generation of immigrants who came to Israel after it was declared a state in 1948. Israeli society has invested tremendous efforts and resources in absorbing these newcomers. However, the scarcity of the state’s means and the acculturation difficulties of these newcomers have contributed to their experiencing substantial suffering (Eliav, 1994). Immigrants have to cope with internal and external expectations for acculturation, that is, with the complicated process of adjusting to and integrating into the society of their new land, as well as with the conflicts between keeping their old traditional ways of living and adopting new habits, norms and ways of thinking (Redfield et al., 1936; Berry and Sam, 1997). Although these immigrants have eventually adjusted and found their place in the Israeli society, it is most likely that throughout this adjustment process, they have sensed that they do not fully belong to the Israeli society, and many of them are likely to wonder to what extent they can regard themselves as Israelis.

It is believed that as far as immigrants are concerned, developing a sense of belonging involves foremost replacing their sense of estrangement by regarding themselves as part of the host society. Furthermore, it is based on believing that members of the absorbing society accept them willingly and consider them an integral part of their community. This aim is not easily obtained. A substantial portion of immigrants who wish to belong is bound to feel only partly socially belonging to this society and only moderately accepted by its members.

Parental and ancestral experiences are absorbed by younger generations (Harper, 2005; Daines et al., 2021). Many cultural practices function to transmit these experiences, especially when they constitute a kind of collective trauma (Bronfenbrenner, 2009). There is reason to assume therefore that the sense of only partial belonging of the first generation of the newcomers to Israel was conveyed to members of the second and third generations, namely their children and grandchildren. The present study investigates the impact of the persistent sense of only partially belonging to one’s society, on the negative and positive coping indicators of the descendants of these immigrants.

### Perceived partial social belonging

Analyses of social integration and belonging often present both constructs in a somewhat dichotomous way. Integration is associated with more positive group affect, whereas segregation is associated with shared negative feelings (Knight and Eisenkraft, 2015). Denial of the opportunity to belong and the feeling of being accepted, valued, and respected (Goodenow and Grady, 1993), is likely to increase negative emotions and decrease the levels of wellbeing and resilience (van Bergen et al., 2019). It is submitted that belonging is a multi-level rather than a dichotomous construct, as people or groups may regard themselves as partly belonging and partly excluded from full participation in a desired social group (Rawal, 2008). In line with previous studies on coping in the context of the COVID-19 pandemic, the significant role of perceived partial social belonging (PPSB) in determining positive and negative coping responses is emphasized. A sense of only partly belonging to a preferred social group promotes higher levels of negative coping indicators and lower levels of positive coping indices (Eshel et al., 2022a,b). It is expected therefore, that a higher sense of PPSB will concurrently decrease levels of resilience and hope and increase anxiety and depression.

### PPSB and demographic variables

Rawal (2008) as well as Eshel et al. (2022b) found that three social groups do not regard themselves as full participants in their society, are bound to have lower resilience and feel considerable distress due to a higher level of PPSB (that was previously referred to as partial social integration–PPSI): younger adults, low-income individuals and females. Younger adults are bound to regard their participation in the mature society as somewhat tentative since they have no way to predict the course of their future lives and are uncertain whether their hopes for the future will come true (Benson and Elder, 2011; Romer and Jamieson, 2020). It has been argued that individuals of low economic standing are more likely to suffer from psychological distress and to sense that they have lower control over their lives than more affluent people (Rodgers et al., 1995). In the last decades, gender roles have changed, and many women have shifted from more traditional to more modern economic and social roles (Esping-Andersen, 2009). Despite these changes, women still play a principal role in managing family life and in caring for their children (Cotter et al., 2011). These responsibilities strongly and often negatively impact their role balance (Holmes et al., 2016).

The extensive research on the need to belong and on social integration has not discussed in depth the gray area that lies between being integrated or segregated, or between feeling of belonging and the sense of not belonging. These research traditions have failed to emphasize the major psychological impacts of PPSB on immigrants, children and youth, as well as on the general public. Our position is that feeling only a partial sense of belonging is sufficient to play a central role in determining the level of positive and negative psychological resources associated with coping of these three social groups with stressful life courses. Furthermore, this study examines the assumption that the importance of PPSB in determining coping with stress will be demonstrated by the finding that it will serve as a mediator between these three social characteristics and both the sense of distress and the resilience of those who are characterized by them.

The present study examines, consequently, PPSB as a mediator of the associations between three stress-evoking demographic characteristics (younger age, low income, and gender) and both lower psychological resilience and higher distress symptoms that are partly caused by these features. Each positive and negative indicator that was assessed in the current study is presented below. All these indicators represent positive psychology concepts (e.g., Oades and Mossman, 2017) that are associated with wellbeing or lack of it and with successful and less successful coping with adversity.

### Positive psychological resources indicators

#### Individual resilience

Masten (2019) defines individual resilience as “the capacity of a system to adapt successfully to disturbances that threaten the viability, function, or development of the system,” indicating that this personal capacity is rooted in and supported by social relationships (Southwick et al., 2016). Individual resilience helps develop a positive coping style and contributes to the advancement of psychological health, a sense of wellbeing (Wu et al., 2020), and physical health (Macía et al., 2021).

Societal resilience is a comprehensive concept concerning the trust in the national authorities, their reliability and social strength, and belief in national cohesion and patriotism (Ben-Dor et al., 2002). Societal resilience was shown to positively associate with varied types of resilience, wellbeing, hope, and morale and negatively associated with anxiety and depressive symptoms as well as perceived perils, during the COVID-19 pandemic (Kimhi et al., 2021).

#### Hope

Snyder (2002) defines hope as “a cognitive set that is based on a reciprocally derived sense of successful goal-directed determination (termed as agency) and planning of ways to meet goals (termed as pathways) (p. 571).” Hope is regarded as a skill aimed at achieving self-management of feelings (Herth, 1992). Greater levels of hope correlate with greater wellbeing and lower psychological distress (Long et al., 2020). Marciano et al. (2022) have demonstrated that in case of danger, hope is the best predictor of the sense of wellbeing, individual and societal resilience, as well as indices of anxiety and depression.

### Negative psychological resources indicators

#### Psychological distress

Psychological symptoms are the most common indicators of the response to threats and disasters. Two substantial distress symptoms are anxiety and depression (Cénat et al., 2020). Research shows that levels of distress negatively affect health regardless of the population studied, the distress tool employed, or the health outcomes examined (Barry et al., 2019).

#### The sense of danger

Threats and disasters often raise a sense of danger. Perceived danger is enhanced when the life or the dignity of the individual or important others are threatened (Eshel and Kimhi, 2016). A higher sense of danger is associated with a lower level of psychological coping (Wang et al., 2020).

The following hypotheses are investigated:

(a) The PPSB level of the whole sample will positively predict the level of distress and sense of danger and negatively predict individual resilience, SR and hope.
(b) Three demographic characteristics will predict the positive and negative coping indices: Younger age, lower income, and female gender will predict lower levels of resilience and hope and higher levels of distress and sense of danger. These demographic variables will negatively predict PPSB, which will mediate their negative prediction of SR, individual resilience, and hope and their positive prediction of distress and a sense of danger.

## Materials and methods

### Data collection

The data were collected online at the responsibility of an internet panel company possessing a database of more than 65,000 residents, representing the varied components of the Israeli society. Israeli individuals (*N* = 1,502) completed a structured survey that required no more than 15 min of their time, between October 12 to 19, 2022. The participants were sampled based on a stratified approach, aligned with the Israeli Central Bureau of Statistics data, appropriately representing the Jewish population’s varied groups (regarding gender, age, religiosity, and family income). Online questionnaires were disseminated to potential participants until the predetermined number of respondents was achieved. All respondents expressed their informed consent to participate in the study, which the Ethics Committee of Tel Aviv University authorized. Furthermore, full anonymity of the respondents was maintained, and no identifying data was given by them. It should be noted, however, that as is the case of other internet panel samples (Mostafa, 2018; Parsaeian et al., 2021), no data is available concerning the response rates of any specific section of this sample, or the reasons of individual respondents for not participating in this study.

### Participants

The needed representative sample of the Israeli Jewish population was calculated to be 385 respondents (OpenEpi). Nonetheless, and in the aim of covering the varied immigration cycles, a large sample of 1,500 respondents was included in the study. The criteria included Jewish respondents, >18 years old. Participants were 1,502 persons from the varied sectors of the Jewish Israeli population (see Table 1). This sample represents wide ranges of income and religiosity levels, political attitudes, and levels of education: The respondents ranged from 18 to 86 years, 50% were women, and 49.9% were men (0.1% defined themselves as “other”). The majority of them (55.3%) reported a family income that was lower than Israel’s average family income, and a somewhat greater majority (58.3%) held right-wing political attitudes. Less than half of the respondents (45.5%) had an academic degree and a similar percentage (45.5%) were secular.

**TABLE 1:** The demographic characteristics of the investigated sample.

Variable scale	Range	Distribution			
		***N***	**%**	**Mean**	**S.D.**
Age	18–30	443	29.5	42.50	15.895
	31–40	297	19.8		
	41–50	280	18.6		
	51–60	237	15.8		
	61+	245	16.3		
Gender	Male	750	49.9		
	Female	751	50.0		
	Other	1	0.1		
Average	Lower	830	55.3	2.38	1.188
Family	Average	395	26.2		
Income	Higher	277	18.5		
**(Scale 1–5)**					
Political	Left	148	9.9	3.59	0.836
Attitude*	Center	447	31.7		
(Scale 1–5)	Right	877	58.3		
Educational	High school	400	26.6	3.33	1.084
Level	Secondary	420	27.9		
(Scale 1–5)	Academia	682	45.5		
Religiosity	Secular	683	45. 5	1.86	0.968
(Scale 1–4)	Traditional	482	32.1		
	Religious	199	13.2		
	Orthodox	138	9.2		

*30 people did not respond to this item.

### Measures

#### Perceived partial social belonging

This scale is based on a previous version employed in research on the COVID-19 pandemic that was named “perceived partial social integration” (Eshel et al., 2022a). The item “Pressures to conform to rules that were determined by others impair my freewill” that was included in the previous version of this scale was replaced by the item “I am uncertain concerning my belonging to the Israeli society.” The scale consists of 7 items about the different components of PPSB (for example: “Despite my achievements I do not feel appreciated socially as much as I deserve to be”). The response scale ranges from 1 (not true at all) to 5 (very much true). According to Cronbach alpha, the reliability of this measure was found to be good (α = 0.83).

#### Individual resilience

The brief two-item Connor-Davidson measure (CD-RISC 2, Campbell-Sills and Stein, 2007) was used to assess personal perceptions of capacity to manage complexities (for example: “I am able to adapt when changes occur”). This measure is assessed by a 5-point response scale ranging from 1 (not true at all) to 5 (generally true). The scale’s reliability according to Cronbach’s alpha was moderate (α = 0.67).

The societal resilience (SR), a 15-item measure developed by Kimhi and Eshel (2019) and Kimhi et al. (2021), evaluates faith in societal leadership (for example: “I have full confidence in the Israeli government’s ability to take the right steps in times of crisis”). The response scale for these items ranges from 1 (strongly disagree) to 6 (strongly agree). The scale’s reliability in the current study was high (α = 0.89).

Hope is a measure that includes 5 items used in former studies (Jarymowicz and Bar-Tal, 2006; Halperin et al., 2008). The items referred to hope in the face of various adversities, such as a pandemic, security threats, or the climate crisis (Example: “I believe that I will be strengthened following each of these crises”). The response scale ranged from 1 (very little hope) to 5 (high hope). The reliability of this 5-item scale was very high (α = 0.92).

Distress symptoms were assessed by nine items of the Brief Symptom Inventory (BSI, Derogatis and Savitz, 2000), representing distress symptoms. The participants expressed the degree of their experiencing the varied symptoms at present (for example: “I feel no hope for the future”). Answers extend from 1 (not at all) to 5 (very much). The reliability of this measure was very high (α = 0.91).

Sense of danger was assessed by 7 items, based on Solomon and Prager’s (1992) scale that referred to the degree of perceived risk at the country, the family, and the personal levels in the current condition of Israel (Example: “To what extent do you feel that your life is in danger?”). The response scale ranges from 1 (Not at all) to 5 (Very much). The reliability of this seven-item scale was high (α = 0.85).

### Statistical analysis

Path analysis (Arbuckle, 2011) was used to investigate the study’s hypotheses controlling for the three demographic predictors (age, gender, and income), the five predicted variables (individual resilience, SR, hope. distress, and sense of danger), as well as the mediating variable (PPSB). The maximal probability assessments were utilized, evaluating a saturated model, as no previous findings were identified that could provide a different model (Arbuckle and Wothke, 2004).

## Results

Examination of Table 2 shows that the mean scores of the psychological resources associated with positive coping were higher than the middle range of their scales. The mean scores of the individual resilience and hope (scales ranging from 1 to 5) are 3.869 and 3.488, respectively, and the mean score of societal resilience (scale ranging from 1 to 6) is 3.464. An opposite direction characterized the coping suppressing variable of distress symptoms (scale ranging from 1 to 5), whose mean score was 2.043. Table 2, presenting the correlations between the investigated variable, shows further that age, family income, gender, and level of education were significantly negatively correlated with PPSB: the lower the age, and the lower the income, the higher the PPSB. In addition, being a woman was related to higher PPSB than men. These findings suggest that younger adults, lower-income individuals, and women tend to regard themselves as only partly integrated into society scoring higher on PPSB. Table 2 indicates that higher level of PPSB is negatively associated with IR, SR and hope and positively and significantly correlated with level of distress and sense of danger. Age and family income were positively correlated with IR, SR and hope, whereas age, family income and level of education were negatively correlated with level of distress and sense of danger.

**TABLE 2:** Pearson correlations between the investigated variables.

Variable	PPSB	IR	SR	Hope	Distress	Danger	Gender	Age	Income	Education
PPSB	1									
IR	-0.175**	1								
SR	-0.382**	0.193**	1							
Hope	-0.212**	0.356**	0.532**	1						
Distress	0.318**	-0.283**	-0.214**	-0.330**	1					
Danger	0.307**	-0.121**	-0.313**	-0.278**	0.519**	1				
Gender	-0.111**	0.001	-0.016	-0.026	0.005	0.080*	1			
Age	-0.317**	0.121**	0.305**	0.119**	-0.194**	-0.208**	-0.090**	1		
Income	-0.197**	0.075**	0.136**	0.054*	-0.084**	-0.229**	-0.143**	0.103**	1	
Education	-0.153**	0.078**	0.030	0.019	-0.076**	-0.103**	0.029	0.219**	0.330**	1
Mean	2.429	3.869	3.464	3.488	2.043	2.506	1.50	42.39	2.38	3.33
S.D.	0.767	0.774	0.859	0.844	0.852	0.864	0.501	15.90	3.19	1.08

*p < 0.05; **p < 0.01.

**FIGURE 1:**
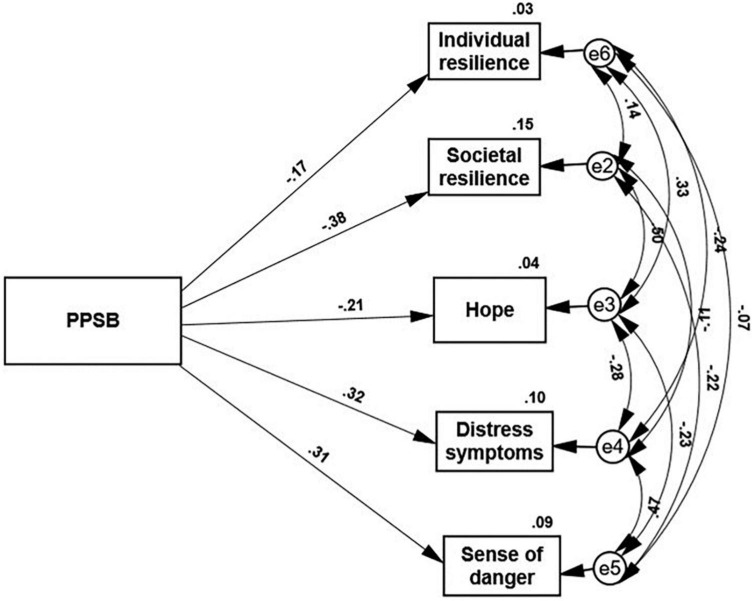
PPSB level predicting coping supporting and coping suppressing personality attributes. All the paths in this figure are significant (*p* < 0.001).

**FIGURE 2:**
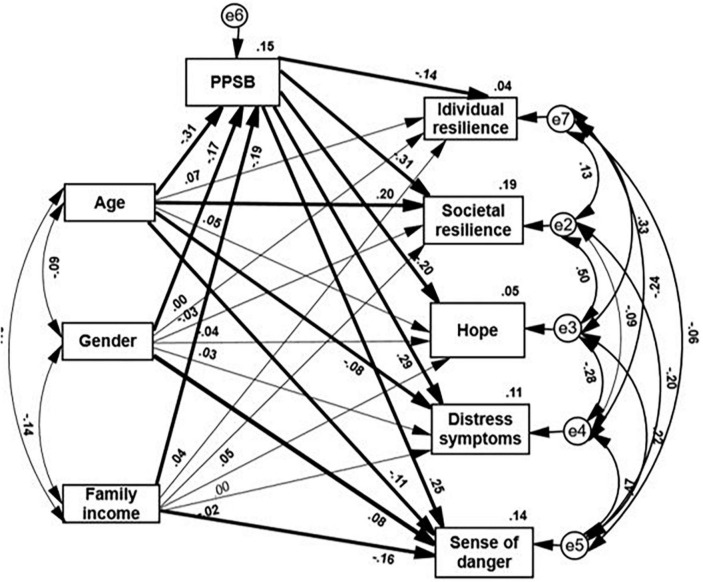
PPSB as a mediator of the associations between three demographic variables and five ensuing psychological characteristics. Paths marked by a thick line indicate a significant path (*p* < 0.05). Standardized direct, indirect, and total effects are significant, *p* < 0.001.

Two path analyses (Figures 1, 2) examined our hypotheses that PPSB will predict the resilience and distress of the whole sample and will mediate the coping responses of the younger and less affluent groups and women. Figure 1 presents the path analysis between PPSB level and coping supporting or suppressing personality attributes that support the first hypothesis. The PPSB level negatively and significantly predicts societal resilience, individual resilience, and hope, and concurrently it positively significantly predicts the levels of distress and sense of danger (*p* < 0.001). PPSB explains 15% of societal resilience, 3% of individual resilience, 4% of hope, 10% of distress, and 9% of sense of danger.

The second path analysis (Figure 2) presents PPSB as a mediator of the associations of age, gender, and family income with the coping supporting and suppressing indices. These results support our second hypothesis. Figure 2 presents a full mediation model. Being a woman, younger age, and having low income, predicted higher levels of PPSB. At the same time, PPSB significantly and negatively predicted social resilience, individual resilience, and hope and positively predicted distress symptoms, and a sense of danger. However, the three independent demographic variables also significantly predicted some of these variables. Sense of danger was significantly predicted by gender (females scored higher), family income (people with lower income scored higher), and age (younger people scored higher). Age also significantly predicted societal resilience and distress: younger people showed lower levels of societal resilience and higher distress symptoms than older individuals.

## Discussion

Belonging is a dynamic construct that refers to being valued and having a sense of fitting in society. This construct is shaped by our perceptions of interactions with people, places, and things (Osterman, 2000). Failure to belong has a range of negative implications for identity, health and wellbeing, academic success, and self-esteem (Morton and Guerin, 2017). This line of research tends to regard belonging and exclusion as a dichotomy and often ignores the intermediate range between these two ends. The present new PPSB construct reveals the more covert aspects of perceived social acceptance and rejection. It emphasizes the major psychological role of a common stressful condition in which individuals perceive themselves as only partially belonging or are not sure to what extent they belong to their society. The present results clearly show that the emotional effects of such PPSB, which do not amount to actual social exclusion, substantially and negatively affect people’s sense of positive coping and increase their coping-suppressing indices.

Reviews of the current research indicate that most of the belonging studies refer to students’ feelings of being accepted, respected, and valued (Parr et al., 2020; Slaten et al., 2020; Allen et al., 2021; Allen et al., 2022). These and other studies show that greater belonging has been consistently associated with more positive psychosocial outcomes. A smaller number of belonging studies referred to the environment that individuals inhabit, such as opportunities to connect in the working place and the sense of space (e.g., Jaitli and Hua, 2013; Trawalter et al., 2020). The distinctive characteristic of the present research is its demonstration that PPSB is not limited to one’s immediate social setting but may actually refer to a much broader social entity, one’s country.

The present study investigates two issues: First, the role of PPSB as a predictor of positive and negative coping indicators in the general Israeli Jewish society. Second, PPSB as a mediator of the associations between three common stress-evoking demographic characteristics (younger age, low income, and gender) and the lower psychological resilience as well as higher distress associated with these features (Eshel et al., 2022b). The results substantiate the role of a new concept of PPSB as an important determinant of social life in general. Figure 1 shows that PPSB negatively predicted societal and individual resilience and hope and positively predicted higher levels of distress symptoms and a sense of danger. Our previous studies (Eshel et al., 2022a,b) demonstrated that PPSB impacts the social behavior of vaccine rejection in the specific case of the COVID-19 pandemic. The present results further indicate that higher PPSB scores are a general social phenomenon that is not limited to particular stressful conditions. Some individuals are likely to sense PPSB in any society, and this feeling is bound to be more prevalent in a society of descendants of immigrants who may be bothered about the extent to which they are accepted as equals by the absorbing society. People may refrain from openly expressing their sense of PPSB. Still, the present data show that this feeling results in substantial psychological and social consequences of lower individual and societal resilience and higher levels of distress. It seems probable that extreme cases of sense of PPSB may threaten the existing social order.

The assumption that PPSB constitutes a common characteristic and an additional distressing factor that impacts groups of people who do not regard themselves as fully belonging to their society, (such as younger adults, low-income persons, and females), was raised in a previous study of the COVID-19 pandemic (Eshel et al., 2022a). The path analysis presented in Figure 2 substantiates this contention, adding that PPSB mediates the association of lower individual and societal resilience and the distress that characterizes each of these three investigated demographic groups.

The present study, which focused on the perception component of Allen et al.’s (2021) model, suggests that perceived partial belonging should be added to the discussion of belonging. PPSB can add refinement to describing the emotions and social perceptions of those who wish to belong but are unsure about their belonging competencies or opportunities and the effect of this uncertainty on their motivation to belong.

### Limitations

The sample employed is substantial and was drawn from an extensive database representing all parts of the Israeli society. However, we have no data concerning the rates of refusals to participate in this study, or the response rates of any specific section of this sample. Thus there is no way to determine the extent to which the present sample constitutes a representative sample of the Israeli-Jewish population. Similar difficulties in obtaining a representative national sample were reported by other researchers (e.g., Mostafa, 2018; Parsaeian et al., 2021).

## Conclusion

The present study presents the new concept of PPSB and demonstrates its relevance to understanding the psychological condition of people who wish to belong, but are unsure whether and to what extent they can regard themselves as an integral part of their society. Previous studies of belonging emphasized more often behaviors and identifications that distinguish those who belong from those who do not belong. Our findings display that the almost ignored condition of being unsure about one’s belonging or regarding oneself as only partly belonging to a desired social group has a major role in increasing psychological distress and sense of danger and in reducing hope and both individual and societal resilience. Moreover, the present data indicate the major role of PPSB in mediating the impact of stress-evoking demographic characteristics on psychological coping.

These findings demonstrate the fact that rather than reflecting an objective condition, social belonging is mainly based on a subjective self-perception of one’s social standing in a desired community. Changing the sense of PPSB seems to be a difficult and prolonged process. Programs aimed at improving the sense of social belonging in any community should start by identifying individuals who are distressed, as they are unsure whether and to what extent they belong to their community. Furthermore, it is and should be believed that the reasons and attitudes that cause PPSB feelings are changeable in the long run.

## Data availability statement

The raw datasets presented in this article are not readily available due to ethical constraints. The analyzed data are available through the authors. Requests regarding the datasets should be directed to the corresponding author.

## Ethics statement

The studies involving human participants were reviewed and approved by the Ethics Committee, Tel Aviv University. The patients/participants provided their written informed consent to participate in this study.

## Author contributions

YE conceptualized the study and drafted the preliminary draft. SK and BA collected the data. YE and SK analyzed the data. HM supervised the study. All authors reviewed the article and approved the final draft.
